# The Association of Mycobacterium tuberculosis with Kikuchi Syndrome: A Case Report and Literature Review

**DOI:** 10.7759/cureus.47075

**Published:** 2023-10-15

**Authors:** Bereket Tewoldemedhin, Nardos Tewoldemedhin, Jack Boghossian, Charity Iheagwara, Alaa Muhanna, Jihad Slim

**Affiliations:** 1 Infectious Diseases, Saint Michael's Medical Center, Newark, USA; 2 Internal Medicine, Addis Ababa University College of Health Sciences, Addis Ababa, ETH

**Keywords:** posttuberculosis kikuchi syndrome, interferon (ifn), cell mediated immune response, lymph node tuberculosis, kikuchi-fujimoto's disease (kfd)

## Abstract

Kikuchi-Fujimoto disease (KFD) is considered one of the rare benign conditions of unknown etiology presenting with the triad of cervical lymphadenopathy, fever, and weight loss. The inciting cause continues to be elusive. One of the leading thoughts is that it may be a post-infectious immune response of T-cells and histocytes. The most common triggers reported have been viral infections. Treatment mainly revolves around the reduction of the inflammatory response with anti-inflammatory medication and steroids when appropriate.

To date, there are very limited reports of *Mycobacterium tuberculosis* as an inciting agent documented. Here, we present a rare case of Kikuchi-Fujimoto disease following *Mycobacterium tuberculosis* infection, more than four years after the completion of therapy.

## Introduction

Kikuchi-Fujimoto disease (KFD), also called histiocytic necrotizing lymphadenitis, was initially reported in Japan in 1972 by Japanese pathologist, Dr. Masahiro Kikuchi, following a case presentation [1]. Dr. Y. Fujimoto independently reported a similar but separate presentation within the same month of that year [1,2]. Although initially seen in young women of Asian descent, it has since been reported across multiple ethnic and racial groups throughout the world [2-4]. The exact mechanism for the development of the disease remains elusive but multiple theories exist predominantly stating either infectious or autoimmune etiology [2,3]. Several studies have identified cytomegalovirus (CMV), Epstein-Barr virus (EBV), varicella-zoster virus (VZV), human herpes virus-6 (HHV-6), parvovirus B19, *Bartonella*, *Toxoplasma*, and *Entamoeba* species from lymph node samples [3,4]. Attempts to establish the causal relationship between the organisms and the disease have largely failed due to the variability of the histologic presentations [4]. 

The mean age of presentation for KFD tends to be less than 40 years of age, but it has been reported in both extremes of age [3,5]. The distribution has a slight predominance in females compared to males in some studies ranging from 1:1.6 to 1:4 of male-to-female ratio of occurrence [3-5].

## Case presentation

A 29-year-old female with a past medical history significant for tuberculosis status post-treatment presents to the emergency department with a complaint of left-sided neck discomfort and swelling worsening over four weeks. The patient stated having otalgia with ear fullness at the outset of symptoms for which she was evaluated by her primary care and was prescribed ofloxacin eardrops along with oral amoxicillin for seven days with minimal improvement. The patient informed noticing a progressive neck swelling while still on the antibiotic and had an erythematous rash that developed around the time of completion of the antibiotics; this involved her upper and lower extremities that spared her face. The rash disappeared after about one week. However, she continued to have progressive enlargement of the left-sided neck swelling associated with redness and discomfort over the area. In addition, she noted low-grade fevers, particularly worse at night, with generalized malaise. She also noted loss of appetite with unquantified weight loss and generalized joint pain with fatigue that prompted her emergency department visit. A review of systems was positive for chills and night sweats for the duration of the symptom onset but negative for cough. 

The patient had immigrated from Morocco to the United States in 2020. She had been diagnosed with left cervical lymph node *Mycobacterium tuberculosis* infection in 2018 while in Morocco after an excisional biopsy was done for an enlargement and fistula formation of the lower left posterior and anterior cervical nodes. At the time, she had received treatment for six months of direct observed therapy followed by six months of treatment for a total 12-month antituberculosis therapy. Her cervical lymphadenopathy had completely regressed at the completion of therapy. She had not traveled back to Morocco since 2020. She had no allergies, no exposure to sick persons, and did not have any pet animals. 

Her vital signs were as follows: temperature 98.1 °C, heart rate 78 beats/minute, blood pressure 113/47 mmHg, respiratory rate 16 breaths/minute, oxygen saturation 98% on room air. The physical exam revealed multiple enlarged, tender, and firm left anterior as well as posterior chain cervical lymph nodes. The largest measured about 1.5 cm in diameter. There was no skin ulceration or discharge present. Laboratory results showed marked leukopenia and elevated erythrocyte sedimentation rate (ESR). Lactate dehydrogenase was elevated. The complete metabolic panel was unremarkable (Table 1).

**Table 1:** Laboratory results of the patient

Parameters	Results	Reference values
Hemoglobin	12.4 g/dl	12-16 g/dl
Platelets	156,000/µL	150,000-400,000/µL
White blood cell	2400/µL	3100-10,500/µL
Neutrophils	1200/µL	1500-7000/µL
Neutrophils (relative percent)	50.5%	40%-60%
Lymphocytes	1000/µL	1000-4000/µL
Lymphocytes (relative percent)	40.2%	20%-40%
Monocytes	200/µL	300-900/µL
Monocytes (relative percent)	7.3%	4%-8%
Lactate dehydrogenase	311 IU/L	120-245 IU/L
Erythrocyte sedimentation rate	20 mm/hour	0 - 20 mm/ hour
C-reactive protein	0.4mg/dl	0.0 - 0.8 mg/dL
Glucose	84 mg/dl	70-140 mg/dl
Blood urea nitrogen	7.0 mg/dl	6.0-24 mg/dl
Creatinine	0.7 mg/dl	0.5-1.0 mg/dl
Sodium	139 mmol/L	136-145 mmol/L
Potassium	4.1 mmol/L	3.5-5.3 mmol/L
Chloride	107 mmol/L	98-110 mmol/L
Albumin	4.2 g/dl	3.6-5.1 g/dl

**Figure 1:**
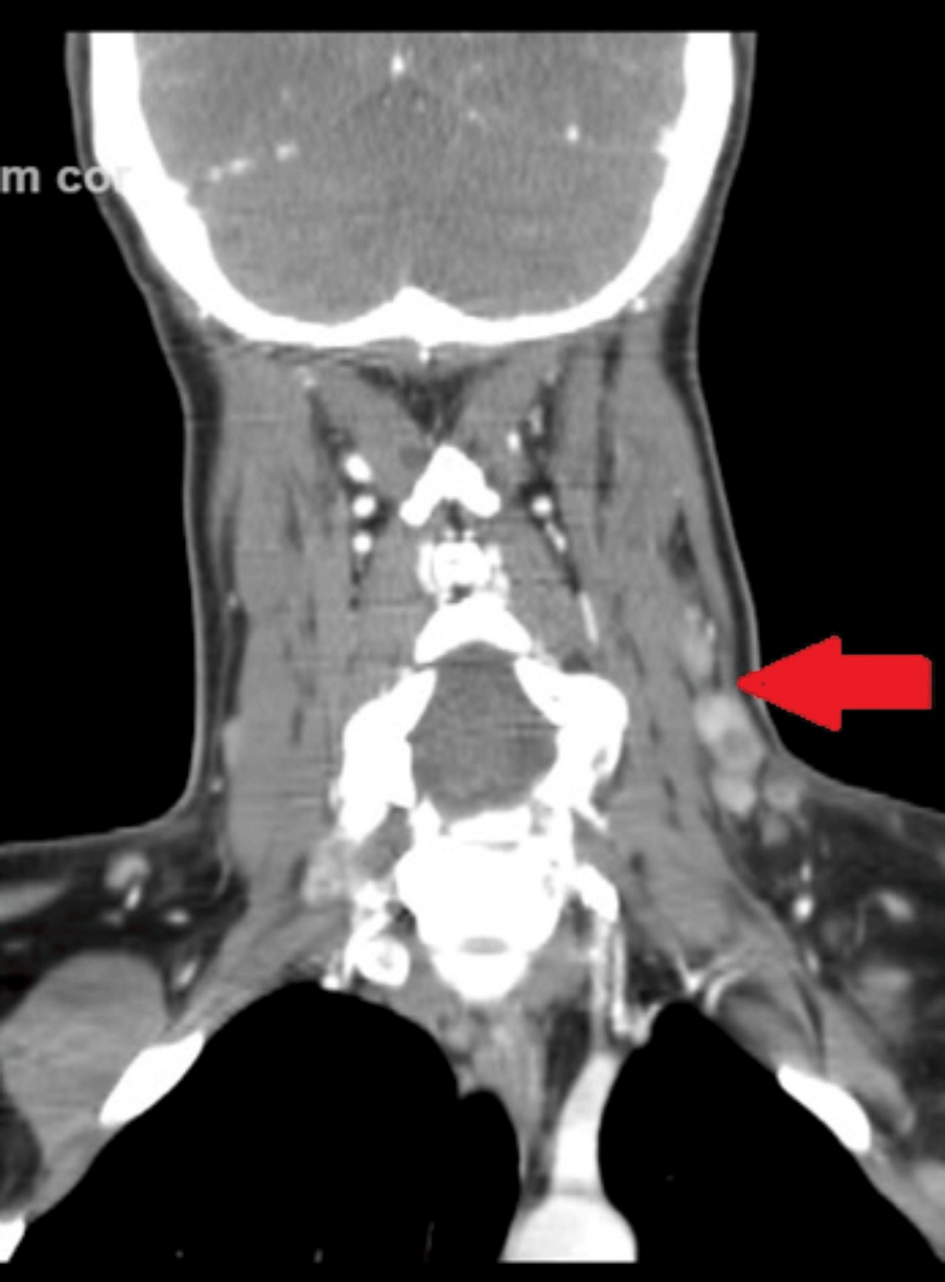
Computer tomography (CT) scan of the neck and soft tissues coronal section revealing multiple lymph node enlargements on the left posterior cervical chain (1.3x1 cm level 1 lymph node, 1x1 cm level 2 lymph node, 1.5x1 cm level 3 lymph node, red arrow)

**Figure 2:**
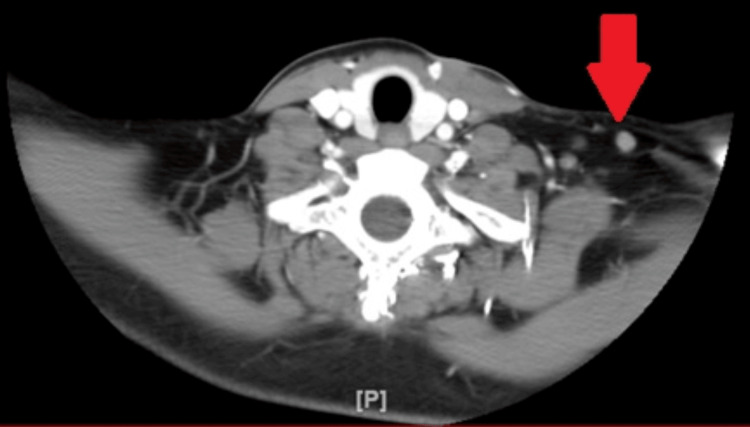
Computer tomography (CT) scan axial section of the neck and soft tissues showing supraclavicular lymphadenopathy (Red arrow)

Serology titers for EBV, CMV, mononucleosis, and HIV, were negative. Anti-nuclear antibody was negative. Bone marrow exam revealed normocellular marrow for age. A computer tomography (CT) scan of her neck and soft tissues revealed multiple lymph node enlargements on the left posterior cervical region (Figure 1) and the supraclavicular regions (Figure 2).

**Figure 3:**
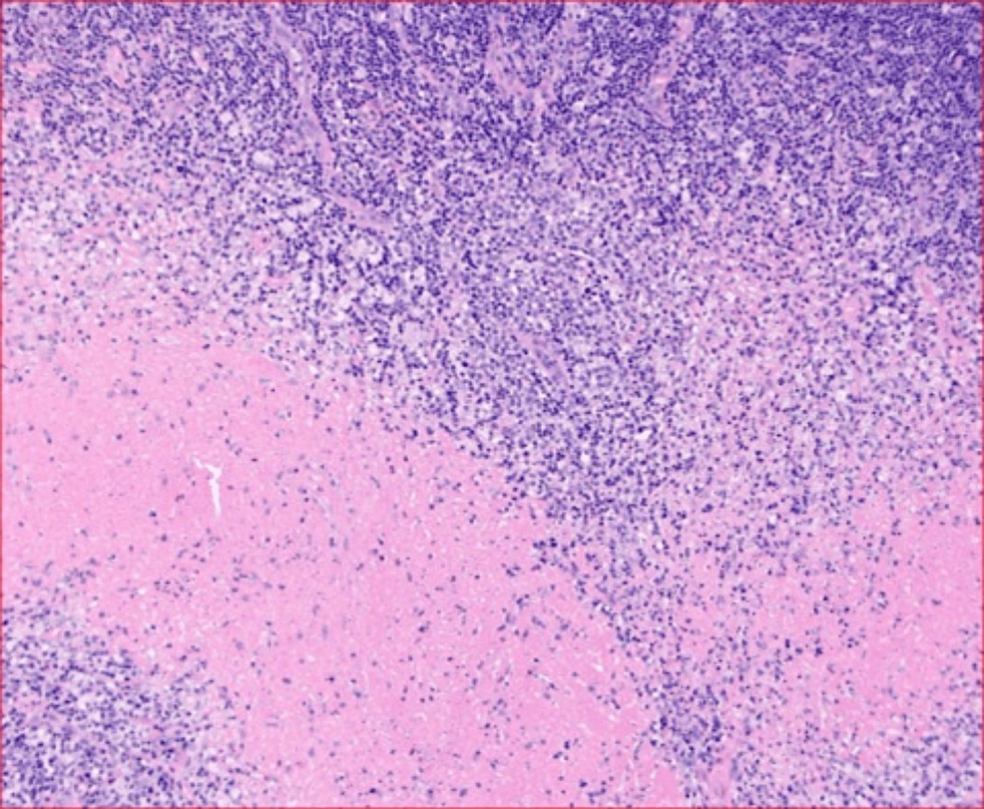
Hematoxylin and eosin stain magnified 100x showing areas of involvement consisting of a central region of necrosis with surrounding zones of mononuclear cells

Blood and sputum cultures were negative. Left neck lymph node excisional biopsy was performed. Acid-fast bacillus (AFB) smear and culture with reflex from the lymph node biopsy site were negative. Fungal cultures from the lymph node biopsy were also negative. Pathology and immunochemistry from the lymph node biopsy of the neck showed an inflammatory lymph node with features of histiocytic necrotizing lymphadenopathy (Figure 3).

**Figure 4:**
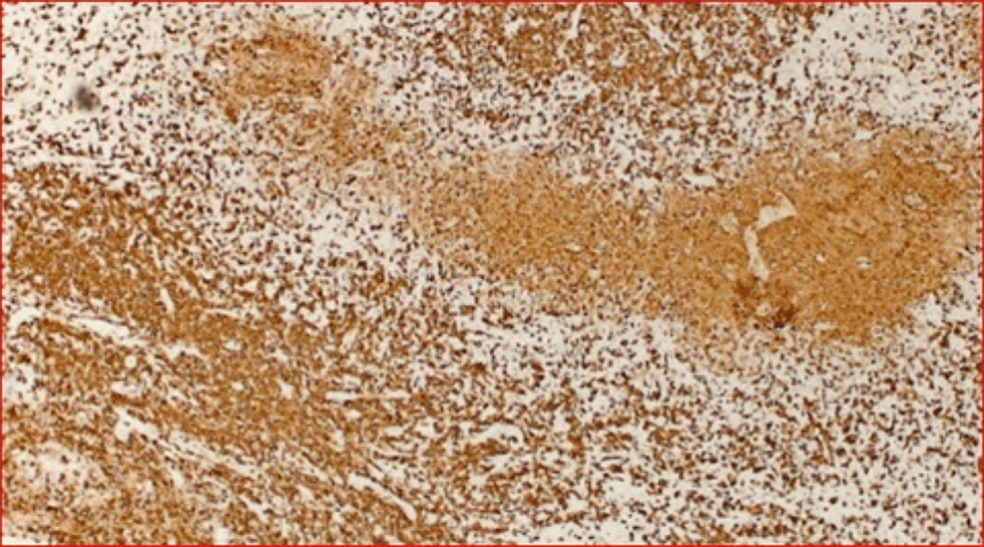
CD3 stain with 40x magnification showing surrounding lymphocytes with predominantly T cells in areas adjacent to the necrosis where histocytes are abundant

Hematoxylin and eosin stain revealed paracortical proliferation of histiocytes and lymphocytes and scattered immunoblasts with necrosis of the area. Numerous histocytes were highlighted by CD68 immunostaining. The majority of the lymphocytes in the lymph node were T-cells, consistent with histiocytic necrotizing lymphadenitis (Figure 4).

The patient was managed with ibuprofen and acetaminophen and discharged. A follow-up at three weeks revealed complete resolution of lymph node swelling and constitutional symptoms. 

## Discussion

Following its initial report in Japan in the 1970s, KFD has since been reported in multiple ethnic as well as racial groups throughout the world [2-4]. The exact mechanism for the development of the disease remains elusive but multiple theories exist predominantly stating either infectious or autoimmune etiology as the underlying factor [3,4]. Several studies have identified CMV, EBV, VZV, HHV-6, parvovirus B-19, *Bartonella*, *Toxoplasma*, and* Entamoeba* species from lymph node samples [4-6]. However, attempts to establish the causal relationship between the organisms and the disease have largely failed due to the variability of the histologic presentation [4,5]. One of the theories in the pathogenesis of KFD is that it is a self-limited immune-mediated inflammatory process triggered by an unidentified, usually infectious, agent that results in the activation of the cytotoxic T cells [6-8]. This theory is supported by the principal histopathologic change observed which includes CD8+ T lymphocyte-mediated cytotoxic apoptosis within the involved lymph nodes with resultant nuclear histocytes phagocytosing apoptotic bodies [5,6,8].

Supporting features that underlying infection plays a role include the transient nature of the disease itself with symptoms resembling an infectious prodrome at its outset [5,6]. The role of interferon-gamma and interleukin-6 in the pathogenesis remains uncertain but elevated serum levels have been noted during active disease which would later trend down during resolution of the disease [6,8]. This is difficult to interpret as it could be a marker of the inflammatory process or can be among the underlying propagating factors. Another emerging theory regarding the etiology of KFD has been an autoimmune inflammatory response [8]. This has been supported by the fact that the patients more likely to develop the disease have shown a higher concentration of human leukocyte antigen (HLA) genes, which indicates some genetic predisposition [6,8]. It has been shown that patients with HLA class II alleles HLA-DPA1 and HLA-DPB1 were more likely to develop the disease. This phenomenon has also been seen to occur with other autoimmune diseases including systemic lupus erythematosus and Sjogren's syndrome [4,6,8].

Clinical manifestations of KFD are often nonspecific resulting in diagnostic difficulty and oftentimes being missed [5]. A study involving more than 100 patients showed that the majority of patients present with fever and lymphadenopathy [6,7]. Other symptoms noted included nonspecific generalized fatigue, arthritis, and a nonspecific erythematous rash seen in a small minority [6,7]. Rare symptoms that have been documented include CNS manifestations with aseptic meningitis, encephalitis, and cerebellar ataxia [6,7]. Commonly encountered laboratory clues included leukopenia seen in approximately 43% of patients with atypical appearing lymphocytes in about 25% [6,7]. Other laboratory findings include anemia, and an elevated ESR in 40% of patients [6,7]. Diagnostic confirmation is obtained from biopsy with histology for lymph node examination showing preserved architecture and follicular hyperplasia with necrotic foci and debris [6,7].

The histologic appearance of biopsied lymph nodes has three stages depending on the disease progress [7]. The early proliferative phase shows follicular hyperplasia paracortical expansion of lymphocytes with histiocytes and numerous apoptosis in the background [7,8]. As the disease progresses through the proliferative stage there is polyclonal infiltration with preservation of the nodal architecture [6-8]. Late stages are characterized by necrotizing infiltrates without neutrophils and a progressive dominance of histocytes as major cell lines [7,8]. The histocytes are predominantly shown to contain phagocytosis debris and are surrounded by abundant CD8+ cytotoxic T cells. There continues to be no consensus regarding treatment for KFD as the disease is mostly self-limiting [6-8]. Patients with mild symptoms respond to supportive therapy with antipyretics [6]. More severe diseases with persistent symptoms can be treated with a short course of high-dose glucocorticoids with the apparent benefit of IV immunoglobulins [6,8]. Multiple recurrences have been reported in a series of 108 patients, of which 92% for whom data was available showed survival past 32 months on follow-up [7,8]. 

Looking at the cellular response to *Mycobacterium tuberculosis* infection, the initial process involves a combination of various pathogen-associated molecular pattern molecule proteins (PAMP) found on the cell wall of the bacilli, which are recognized by the toll-like receptor (TLR)-2 on the surface of macrophages [9,10]. This induces an intense proinflammatory response with an expression of tumor necrosis factor-alpha and various interleukins including IL-6 and IL-12 [9,10]. Once adhesion and phagocytosis of the bacilli take place, a complex series of events are created by the bacillary cell wall glycolipid lipoarabinomannan to prevent phagolysosome-mediated destruction. Among the strategies is inhibition of the production of the phosphatidylinositol 3-phosphate, which normally is responsible for the destruction of bacteria within the phagolysosome [10]. This results in the arrest of bacillary destruction with replication of the bacilli within the macrophages eventually rupturing and infecting other macrophages [10,11]. This unchecked replication results in extensive growth of the bacteria within the naïve macrophages and granuloma formation [10,11]. Studies have shown that the proinflammatory reaction created by the dysregulated signaling of the immune system due to the constant cell lysis and re-infection allows expansion of the granuloma with bacterial proliferation [9-11]. Approximately two to four weeks after infection, two host responses develop. The first is macrophage activating cell-mediated immune (CMI) response which is a T-cell-mediated phenomenon [10,12]. This is caused by the migration of the macrophages and dendritic cells to draining lymph nodes and presentation to the T lymphocytes. This in turn activates the macrophages capable of killing the bacilli [10-2]. The other response is a tissue damage response resulting from a delayed-type hypersensitivity response to the various bacillary antigens [10-12]. This destroys the dormant macrophages bacilli complex [11,12]. The result of all this is the caseating granuloma classically seen in *Mycobacterium tuberculosis* infection.

The CMI is critical in the early stages and does confer partial protection against *Mycobacterium tuberculosis* infection [9,12,13]. CD4+ T cells play a crucial role in the production of Interferon-gamma, which is an essential chemokine for macrophage activation and chemotaxis [11,12]. CD8+ T cells have also been shown to play an important role and are upregulated during the infection [11,12]. They have been associated with protective activities by promoting the lysis of infected cells and upregulation of interferon‐gamma (IFN‐γ) [9,11,12]. Several observations have shown that certain genetic factors play a key role in innate nonimmune resistance as well as the progression of the disease of *Mycobacterium tuberculosis* [12,13]. Among susceptibility factors, polymorphisms in genes that encode HLA alleles have been postulated with rapid progression of disease [12-14]. Genes that code for the natural resistance-associated macrophage protein 1, which is present on chromosome 2q, have been shown to confer some resistance against the rapid development of *Mycobacterium tuberculosis* disease [13,15]. 

There are multiple overlaps of the immune responses seen with *Mycobacterium tuberculosis* infection and KFD. The role of Interferon-gamma in both seems to be among the crucial responses in the cytokine release. Further, the presence of a predominantly T-cell response in *Mycobacterium tuberculosis* infection may serve as a predisposition to the pathogenesis of KFD as it incites a very similar immunologic response. The genetic susceptibility pattern associated with HLA polymorphism in both disease entities indicates there is an undercurrent of similarity in the pathogenesis of both diseases. 

An extensive literature search was done on the PubMed database for English articles published between 1993 and 2023 related to KFD in patients after *Mycobacterium tuberculosis* infection. The terms "Kikuchi Fujimoto disease" and "*Mycobacterium tuberculosis*" were used as keywords. Screening references of retrieved articles identified 35 publications. In six publications, *Mycobacterium tuberculosis* was in the differential or worked up. One paper that studied the major clinical presentations of patients who underwent biopsy of the cervical lymph nodes with histologically proven KFD between 2006 and 2008 involving a total of 30 patients noted that two of the subjects had a past history of *Mycobacterium tuberculosis*; however, no further details as to the treatment was provided [16]. 

To the best of our knowledge, there have been no reported cases of this disorder following therapy for *Mycobacterium tuberculosis* infection. Several studies have shown that *Mycobacterium tuberculosis* infectioncan often be the first diagnosis in patients with KFD and as a result, patients have been subjected to unnecessary antituberculosis therapy before the diagnosis of KFD is made [17-20]. 

The major limitation in our patient’s presentation setting has been the limited data available on the exact pathological findings during the initial diagnosis of her *Mycobacterium tuberculosis* infection. This was compounded by the scarcity of cases reported with a similar presentation timeline.

## Conclusions

KFD is a rare entity and, as a result, there is a significant gap in understanding the etiology and pathophysiology. There seems to be an extensive overlap between genetic susceptibility and the immunologic response to *Mycobacterium tuberculosis *infection. Further studies are needed to elucidate whether there is a causal relationship between the two distinct disease entities. Early identification of the risk factors for KFD can help better understand the disease and provide significant improvement in the morbidity associated with it. This case report aims to highlight the importance of the need for further studies to elucidate the pathophysiology of KFD and pave the discussion for further research. 
